# Taurine Augments Telomerase Activity and Promotes Chondrogenesis in Dental Pulp Stem Cells

**DOI:** 10.3390/jpm11060491

**Published:** 2021-05-31

**Authors:** Mohammed Mashyakhy, Ahmed Alkahtani, Abdulaziz S. Abumelha, Reham Jamal Sharroufna, Mazen F. Alkahtany, Mohamed Jamal, Ali Robaian, Sultan Binalrimal, Hitesh Chohan, Vikrant R. Patil, A. Thirumal Raj, Shilpa Bhandi, Rodolfo Reda, Luca Testarelli, Shankargouda Patil

**Affiliations:** 1 Department of Restorative Dental Sciences, College of Dentistry, Jazan University, Jazan 45412, Saudi Arabia; dr.mashyakhy@gmail.com (M.M.); drhiteshchohan@yahoo.co.in (H.C.); shilpa.bhandi@gmail.com (S.B.); 2 Department of Restorative Dental Sciences, Division of Endodontics, College of Dentistry, King Saud University, Riyadh 11451, Saudi Arabia; ahkahtani@ksu.edu.sa (A.A.); Malkahtany@ksu.edu.sa (M.F.A.); 3 Department of Restorative Dental Sciences, College of Dentistry, King Khalid University, Abha 61421, Saudi Arabia; aabumelha@kku.edu.sa; 4 Private Hospital, Khobar 122001, Saudi Arabia; reham.sharroufna@hotmail.com; 5 Department of Endodontics, Hamdan Bin Mohamed College of Dental Medicine, Mohammed Bin Rashid University of Medicine and Health Sciences, Dubai Health Care City, Dubai 66566, United Arab Emirates; mohamed.jamal@mbru.ac.ae; 6 Conservative Dental Sciences Department, College of Dentistry, Prince Sattam Bin Abdulaziz University, Alkharj 11942, Saudi Arabia; ali.alQahtani@psau.edu.sa; 7 Restorative Department, College of Dentistry, Riyadh Elm University, Riyadh 12611, Saudi Arabia; Sultan@riyadh.edu.sa; 8 Biogenre Private Limited, Pune 412105, India; patilvikrant.r@gmail.com; 9 Department of Oral Pathology and Microbiology, Sri Venkateswara Dental College and Hospital, Chennai 600130, India; thirumalraj666@gmail.com; 10 Department of Oral and Maxillofacial Sciences, Sapienza University, University of Rome, 00185 Rome, Italy; rodolforeda17@gmail.com (R.R.); luca.testarelli@uniroma1.it (L.T.); 11 Department of Maxillofacial Surgery and Diagnostic Sciences, Division of Oral Pathology, College of Dentistry, Jazan University, Jazan 45412, Saudi Arabia

**Keywords:** mesenchymal stem cells, chondrogenesis, telomerase, taurine, dental pulp, regenerative medicine

## Abstract

Background: Stem cell therapy has become an advanced and state-of-the-art procedure to regenerate lost tissues of the human body. Cartilage repair is a challenging task in which stem cells find potential application. One of the important biologic modifiers that can cause chondrogenic differentiation of stem cells is taurine. However, taurine has not been investigated for its effects on dental pulp derived stem cell (DPSC) chondrogenic differentiation. Objective: The objective of the study was to investigate if taurine administration to DPSCs heralds chondrogenic differentiation as ascertained by expression of SOX9, COL2A1, ACAN, ELN, and COMP. The study also investigated if the differentiated cells synthesized glycosaminoglycans, a marker of cartilage formation. The study also aimed to assess proliferative activity of the cells after taurine administration by measuring the hTERT gene and protein expression. Materials and methods: DPSCs were obtained from a molecular biology laboratory and characterization of stem cell markers was done by flow cytometry. The cells were subjected to a MTT assay using various concentrations of taurine. Following this, hTERT gene and protein estimation was done in the control, telomerase inhibitor treated DPSC (TI-III), 10 μM taurine treated DPSC, and TI-III + 10 μM taurine treated DPSCs. A polymerase chain reaction was done to assess gene expression of SOX9, COL2A1, ACAN, ELN, and COMP genes and glycosaminoglycans were estimated in control cells, Induced DPSCs, induced and TI-III treated DPSCs, and 10 μM taurine treated DPSCs. Results: DPSCs expressed CD73, CD90, and CD105 and did not express CD34, CD45, and HLA-DR, which demonstrated that they were mesenchymal stem cells. The MTT assay revealed that various concentrations of taurine did not affect the cell viability of DPSCs. A concentration of 10 μM of taurine was used for further assays. With regard to the hTERT gene and protein expression, the taurine treated cells expressed the highest levels that were statistically significant compared to the other groups. Taurine was also found to restore hTERT expression in telomerase inhibitor treated cells. With regard to chondrogenesis related genes, taurine administration significantly increased the expression of SOX9, COL2A1, ACAN, and ELN genes in DPSCs and caused a significant increase in glycosaminoglycan production by the cells. Conclusions: Taurine can be regarded a biologic modifier that can significantly augment chondrogenic differentiation of DPSCs and can find potential applications in regenerative medicine in the area of cartilage regeneration.

## 1. Introduction

Cartilage degeneration is associated with severe morbidity due to the limited regenerative capacity, repair, and the current availability of therapeutic strategies [1]. Recent research has led to the innovation of autologous chrondrocyte therapy wherein autologous chrondrocytes are cultured and expanded in vitro and are injected into the target site either as a cell suspension or with a matrix (MACT), which will aid in cartilage regeneration [2,3]. However, autologous chondrocyte therapy has several limitations such as injury of healthy cartilage during surgery, which frequently results in fibrocartilage formation at defective sites. It is also to be understood that regardless of the type of cells used for transplantation therapy, the scaffold used as a carrier for the cells plays a pivotal role in the outcome of the procedure. Additionally, it would be appropriate to choose a good scaffold with optimum regenerative properties and load the cells of interest onto it. In this regard, stem cells have been explored for the regeneration of several tissues [4]. In vivo stem cell transplantation in animal models have demonstrated that these cell populations can aid in regenerating structure and restoring function by a plethora of complex mechanisms [5,6,7]. The different types of stem cells include embryonic stem cells (ESCs), fetal stem/progenitor cells, induced pluripotent stem cells (iPSCs), and adult stem cells. Although embryonic stem cells and induced pluripotent stem cells possess differentiation potential, they also have a risk for tumorigenesis [8]. Although adult stem cells have a lower proliferation rate and differential potential, they have a relatively safer record in clinical application, and further ethical concerns are also minimal [8,9,10,11,12].

The major limitation in applying mesenchymal stem cells (MSCs) for the repair of articular cartilage is to regenerate cells with stable chondrocyte characteristics capable of resisting hypertrophy and terminal differentiation. Although in vitro protocols for differentiating MSCs into chondrocytes have been successful, in vitro chondrogenesis of MSCs have been shown to exhibit additional capability such as promoting the induction of fibrocartilage-like features mediated through collagen type I expression and hypertrophy.

Dental stem cells are a specific type of mesenchymal stem cells sourced from the dental pulp, apical papilla, periodontal ligament, and dental follicle [13,14]. Among the various sources of dental stem cells, pulp is a very important source as derived from the neural crest and possesses the property of self-renewal and differentiation into several cell lineages [13]. Recently, mechanical factors along with biochemical factors have been recognized to serve as primary regulators of the behavior and function of DPSC. Several forces such as uniaxial mechanical stretch, pulsating fluid flow, low-intensity pulsed ultrasound influence the DPSCs and their properties [15]. The specific surface markers expressed by DPSCs are similar to the markers expressed by embryonic stem cells (ESCs) and MSCs [13,16,17]. Hence, DPSCs are currently being explored in regenerative medicine. The other advantage of DPSCs include limited ethical considerations [18]. Thus, DPSCs could be explored for cartilage repair. It is recently understood that the secretome of stem cells contain plenty of growth factors that can enrich not only these cells, but also contribute to the differentiation and proliferation of a variety of other cell types. This concept of using the secretome of stem cells with biomaterials, growth factors, and scaffolds is currently documented with results in enhanced regeneration and repair [19].

Growth factors such as TGF-*β,* BMP-2, -4, -6, -7, -13, and -14, IGF1, FGF2, are known to stimulate chondrogenesis of stem cells. In this regard, 2-aminoethanesulfonic acid or taurine, a sulfur containing β-amino acid commonly seen in animal tissues, is being explored for its chondrogenic stimulation potential. The major source of taurine is diet and the minor source is endogenous synthesis from methionine and cysteine in the presence of vitamin B_6_ [20]. The various biological functions of taurine include the formation of bile salt, lipid metabolism, antiatherogenic effects, homeostasis of sodium, calcium and neurotransmitters, protective role against ischemia-reperfusion injury, antioxidant, antihypertensive, antianxiety, management of alcoholism, fatigue and myotonia, improves diabetic vision impairment, antitumor, anti-inflammatory, and anti-neurotoxic action [21,22,23,24,25,26,27,28,29,30,31,32,33,34,35,36,37,38]. Studies have demonstrated that taurine promotes chondrocyte proliferation and chondrocyte differentiation in human umbilical cord derived stem cells [39,40]. However the effect of taurine on chrondrogenic differentiation of DPSCs has not been reported thus far. It has also been demonstrated that administration of vitamin C to periodontal ligament stem cells (PDLSC) augments the formation of cell sheets and upregulates telomerase expression and consequently enhances osteogenic differentiation. Whether taurine can exert similar effects like vitamin C is largely unknown [41]. Hence, with the available information, the present study was conducted to assess the effect of taurine on the telomerase activity and chondrogenesis in dental pulp stem cells.

## 2. Materials and Methods

### 2.1. Source and Cell Culture Protocol for Dental Pulp Stem Cells

Scientific research (IRB)-College of Dentistry, Jazan University approved the present study (Ref. no. CODJU-19714). After obtaining the informed consent, DPSCs were isolated from a healthy permanent tooth (age: 14–25 years) extracted for orthodontic purposes with appropriate oral hygiene (*n* = 3) by using the explant culture method described previously [42]. Pulp tissue was macerated into tiny pieces and were transferred to 35 mm polystyrene plastic culture Petri dishes. An adequate amount of fetal bovine serum (FBS) (Gibco, Rockville, MD, USA) was added to the tissues to cover them completely. A 24 h incubation protocol was performed at 37 °C and 5% CO_2_ for the explants immersed in FBS; the DPSCs were further expanded in DMEM (Invitrogen, Carlsbad, CA, USA) supplemented with 20% FBS and antibiotic-antimycotic at the ambient temperature and CO_2_ condition. The culture medium was replenished once in two weeks, and the proliferation and morphological characteristics were ascertained regularly with an inverted phase-contrast microscope. After 70–80% confluence was attained, the cells were detached using 0.25% Trypsin-EDTA solution (Invitrogen, Carlsbad, CA, USA) and transferred to a bigger 25-cm^2^ polystyrene culture flask (Nunc, Rochester, NY, USA). Cells from passage 2 to 4 were used for all the experiments.

### 2.2. Flow Cytometric Characterization of DPSCs

The cells were subjected to trypsinization and washed twice with phosphate buffered saline (PBS) and incubated for half an hour at 4 °C with anti-human-CD73-APC, anti-human-CD90-APC, anti-human-CD105-APC, anti-human-CD34-PE, anti-human-CD45-FITC, and anti-human-HLA-DR-APC antibodies (Miltenyi Biotec, Bergisch-Gladbach, Germany). The stained cells were washed twice with PBS. Following this, ten thousand cells per sample were acquired on a FACS Canto II flow cytometer (BD Biosciences, San Jose, CA, USA). Isotype control was used for differentiation of positive and negative signals.

### 2.3. Osteogenic Differentiation

A count of 2500 cells/cm^2^ was seeded in a 24-well plate system (Nunc, Rochester, NY, USA) with complete growth medium. After 24 h, medium was replaced with osteogenic induction medium, which was DMEM supplemented with 1% antibiotic-antimycotic, 0.1 µM of dexamethasone, 50 µM of ascorbate-2-phosphate, and 10 mM of β-glycerophosphate (Sigma-Aldrich Corp., St. Louis, MO, USA). The medium was frequently replaced once a week. To analyze the osteogenesis in the cells after 21 days, fixing was performed with 4% paraformaldehyde, and the 2% alizarin red S (pH 4.1–4.3) staining protocol was performed for a time period of 20 min.

### 2.4. Adipogenic Differentiation

A total of 2500/cm^2^ of cells were seeded onto a 24 well plate following a cell counting protocol (Nunc, Rochester, NY, USA) and immersed with complete growth medium. After 24 h, the medium was removed and adipogenic media were added (DMEM supplemented with 10% FBS, 1 µM dexamethasone, 10 µM insulin, 200 µM indomethacin, and 0.5 mM isobutyl-methylxanthine (Sigma-Aldrich Corp., St. Louis, MO, USA) and the cells were exposed to the media twice a week for three weeks. Three experimental groups were formed: control, induction, and 5 µM cordycepin treatment with induction. Differentiated adipocytes were treated with 4% paraformaldehyde and stained with 0.3% Oil Red O for oil droplets for 1 h.

### 2.5. Chondrogenic Differentiation

A standard count of 2500 cells/cm^2^ was seeded onto a 24-well plate (Nunc, Rochester, NY, USA) with complete growth medium. After a 24-h period, the complete growth medium was replaced with a chondrogenic medium to initiate induction, which was DMEM with 1X-ITS, 1 mM of sodium pyruvate, 100 nM of dexamethasone, 50 µg/mL of ascorbate-2-phosphate, 40 µg/mL of L-proline, and 10 ng/mL of TGF-β3 (Sigma-Aldrich Corp., St. Louis, MO, USA). Cultures were maintained for a period of 28 days at 37 °C in a 5% CO_2_ incubator and the medium were replenished with fresh medium every 2–3 days. For analysis of chondrogenic differentiation, Alcian blue staining was performed on fixed cells after 21 days. Cells were fixed with 4% paraformaldehyde and stained with 0.1% Alcian blue for 30 min. Experimental groups are given in Table 1.

### 2.6. Cytotoxicity Assay for Determination of Effect of Taurine on DPSCs

The MTT assay was performed to assess the effect of taurine on the cell viability of DPSCs. The cells were seeded in 96-well plates at a density of 5 × 10^3^ cells in each well and treated with various concentrations of the taurine (Tau) (Sigma Aldrich, St. Louis, MO, USA) (1 µM, 2.5 µM, 5 µM, 10 µM, 25 µM, and 50 µM) and incubated for 48 h and seven days (media was replaced with fresh appropriate media twice, taurine treatment was also repeated with media replacement for seven days). Following this, MTT solution (Sigma-Aldrich Corp., St. Louis, MO, USA) at a concentration of 0.5 mg/mL was added to each well and mixed. Following a four-hour incubation at 37 °C, the medium was removed, and 100 µL dimethyl sulfoxide (DMSO) (Sigma-Aldrich Corp., St. Louis, MO, USA) was added to each well. The absorbance was measured at 570 nm using a Multiskan Spectrum spectrophotometer (Thermo Scientific, San Jose, CA, USA).

### 2.7. Determination of the Effect of Taurine on Chondrogenic Differentiation of DPSCs

The cells were seeded at the density of 2500 cells/cm^2^ in a 24-well plate (Nunc, Rochester, NY, USA) with complete growth medium. After 24 h, the complete growth medium was replaced with a chondrogenic induction medium, comprising of DMEM with 1X-ITS, 1 mM of sodium pyruvate, 100 nM of dexamethasone, 50 µg/mL of ascorbate-2-phosphate, 40 µg/mL of L-proline, and 10 ng/mL of TGF-β3 (Sigma-Aldrich Corp., St. Louis, MO, USA). Four experimental groups were created: control, induction, telomerase inhibitor III (Sigma Aldrich, St. Louis, MO, USA) + induction, and 10 μM taurine (Tau). Cultures were incubated for seven days, 14 days, and 21 days at 37 °C in a 5% CO_2_ incubator; the medium was replaced with a fresh medium every 2–3 days. For the control group, the cells were incubated with the plain growth medium alone. For analysis for differentiation toward chondrogenic lineage, cells were fixed with 4% paraformaldehyde and stained for glycosaminoglycans using 0.1% toluidine blue [43]. The toluidine blue intensity was quantified by dissolving stained cells in 4% acetic acid, and the absorbance was measured colorimetrically at 450 nm. For Alcian blue staining, cells were fixed with 4% paraformaldehyde and stained with 0.1% Alcian blue for 30 min. For safranin O staining, cells were fixed with 10% formalin for 30 min, then, 0.1% safranin O was added to the cells for 30 min.

### 2.8. Real-Time Quantitative PCR for Analysis of Gene Expression

DPSCs were seeded into 12-well plates at the cell density of 1 × 10^5^ cells per well and incubated for 24 h. For human TERT gene expression analysis, four experimental groups were created: the control, TI-III, 10 μM Tau, and TI-III + 10 μM Tau (Table 2). Cultures were incubated for seven days at 37 °C in a 5% CO_2_ incubator; the medium was replaced with a fresh medium twice. For chondrogenesis related gene expression analysis, four experimental groups were created: the control, induction, TI-III + induction, and 10 μM Tau (Table 1). Cultures were incubated for 21 days at 37 °C in a 5% CO_2_ incubator; the medium was replaced with a fresh medium every 2–3 days. The total RNA was extracted from the cells by using the GeneJET RNA Purification Kit (Thermo Scientific, Vilnius, Lithuania). RNA (2 μg) was reverse transcribed using a cDNA Synthesis Kit (High Capacity, Applied Biosystems, Carlsbad, CA, USA) according to the manufacturer’s guidelines. Total of 100 ng cDNA was used for the total reaction volume of 20 μg for each gene. Quantitative analysis of genes of interest was carried out using the SYBR Green PCR master mix (Applied Biosystems, Austin, TX, USA) on a Real-Time PCR system (QS5, Applied Biosystems, Foster City, CA, USA). Expressions of target genes related to stemness, pluripotency, and differentiation were normalized to GAPDH as a reference gene using the ΔΔCt method [44]. The list of genes and primers (Eurofins) are given in the Table 3.

### 2.9. Western Blot Analysis

DPSCs were seeded into 12-well plates at the cell density of 1 × 105 cells per well and incubated for 24 h. For hTERT protein expression analysis, four experimental groups were created: the control, TI-III, 10 μM Tau, and TI-III + 10 μM Tau. Cultures were incubated for seven days at 37 °C in a 5% CO_2_ incubator; the medium was replaced with a fresh medium twice. The cells were lysed using RIPA buffer (Sigma Aldrich, St. Louis, MO, USA) and the lysates were centrifuged at 12,000 rpm for 15 min. The supernatant was collected, and the protein content was determined using the protein assay. SDS-PAGE sample buffer was introduced to the lysates and the lysates were heated to 100 °C for 8 min. Of the total protein, 20 μg was loaded in each well of a 10% SDS-PAGE gel on Mini-PROTEAN Tetra Cell (Bio-Rad Laboratories Inc., Hercules, CA, USA). Western blot technique was performed by using hTERT antibody and GAPDH antibody (Abcam, Cambridge, UK) as primary antibodies and HRP-conjugated secondary were used for the detection of proteins of interest.

### 2.10. Immunofluorescence Assay

Cells were treated with 4% paraformaldehyde for 20 min and further blocking was done with 20% FBS in PBS for 20 min, at room temperature. Three rounds of PBS wash was performed following each step. Fluorophore tagged antibodies SOX-9-PE and aggrecan-FITC (BD Pharmingen) were added at appropriate concentrations in PBS and incubation was performed at 4 °C for 3 h. The cells were washed three times with 1× PBS and counterstaining with 1:10,000 DAPI for 2 min was undertaken. After washing with 1× PBS, the cells were observed under an Olympus IX73 inverted microscope and images were captured at 20× magnification.

### 2.11. Statistical Analysis

Each experiment was replicated in triplicate. The results were shown as the mean ± standard deviation of the values from the three independent experimental values. The data were analyzed by using the unpaired *t*-test (two-tailed) and *p* < 0.05 was considered as significant (ns not significant, * *p* < 0.05, and ** *p* < 0.01).

## 3. Results

DPSCs show characteristic marker expression for MSC-specific markers.

**Figure 1:**
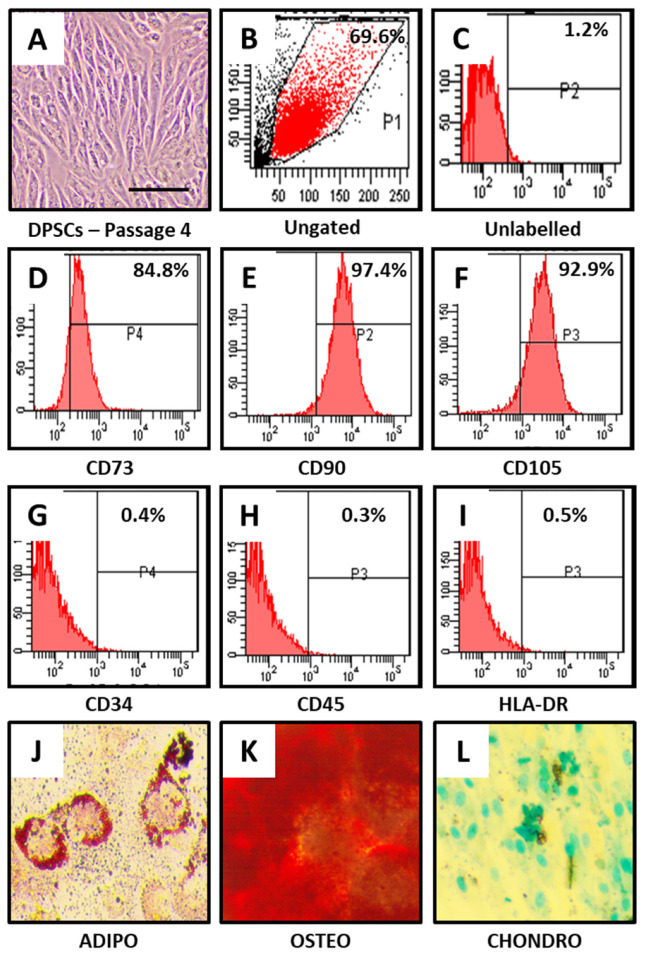
Characterization of DPSCs for mesenchymal stem cell (MSC) markers and trilineage differentiation. (**A**) Photomicrograph of DPSCs at passage 4. (**B**–**I**) DPSCs were checked for MSC-specific positive markers CD73, CD90, and CD105; and MSC-specific negative markers CD34, CD45, and HLA-DR. (**J**–**L**) DPSCs were differentiated into adipocytes, osteoblasts, and chondrocytes. Scale bar = 100 μm. DPSCs: dental pulp stem cells, ADIPO: adipogenic differentiation, OSTEO: osteogenic differentiation, CHONDRO: chondrogenic differentiation.

DPSCs showed MSC-like morphology (Figure 1A) and positive expression for MSC-specific markers. CD73, CD90, and CD105 showed positive expression in DPSCs (Figure 1B–F), whereas non-MSC markers CD34, CD45, and HLA-DR showed negative expression in DPSCs (Figure 1G–I).

Furthermore, DPSCs were checked for their trilineage differentiation aptitude. It was observed that DPSCs were able to differentiate into adipocytes, osteoblasts, and chondrocytes as observed by functional staining methods (Figure 1J–L).

Taurine at higher concentrations affected the viability of DPSCs after long-term exposure.

**Figure 2:**
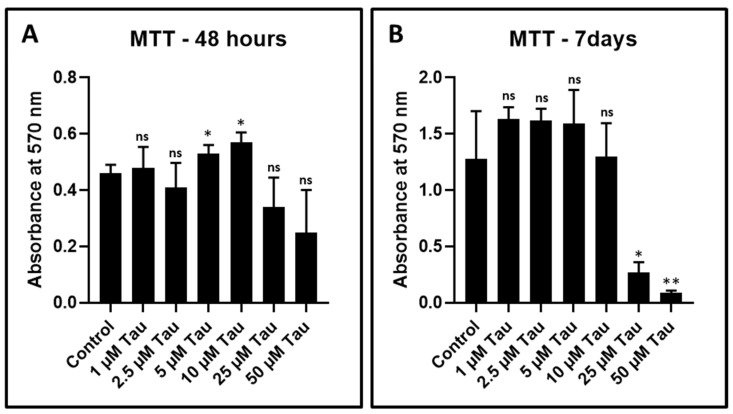
MTT assay to assess the cytotoxicity of taurine to DPSCs. (**A**,**B**) DPSCs were treated with various concentrations of taurine (1 μM, 2.5 μM, 5 μM, 10 μM, 25 μM, and 50 μM) for 48 h and seven days and comparative analysis was done to check the cytotoxicity of taurine to DPSCs. ns: not significant, * *p* < 0.05, ** *p* < 0.01. Tau: Taurine.

DPSCs were treated with various concentrations of taurine (1 µM, 2.5 µM, 5 µM, 10 µM, 25 µM, and 50 µM). Cell viability was assessed by the MTT assay after 48 h and seven days of treatment. None of the concentrations showed any significant decrease in the viability of DPSCs at 48 h (Figure 2A and Table 4). However, after 7 days, treatment with taurine exerted a cytotoxic effect on DPSCs at 25 µM and 50 µM concentrations (Figure 2B). A total of 10 µM taurine was used for further experimentation.

Taurine treatment augments the expression of the TERT gene and triggers protein production of telomerase reverse transcriptase (hTERT).

**Figure 3:**
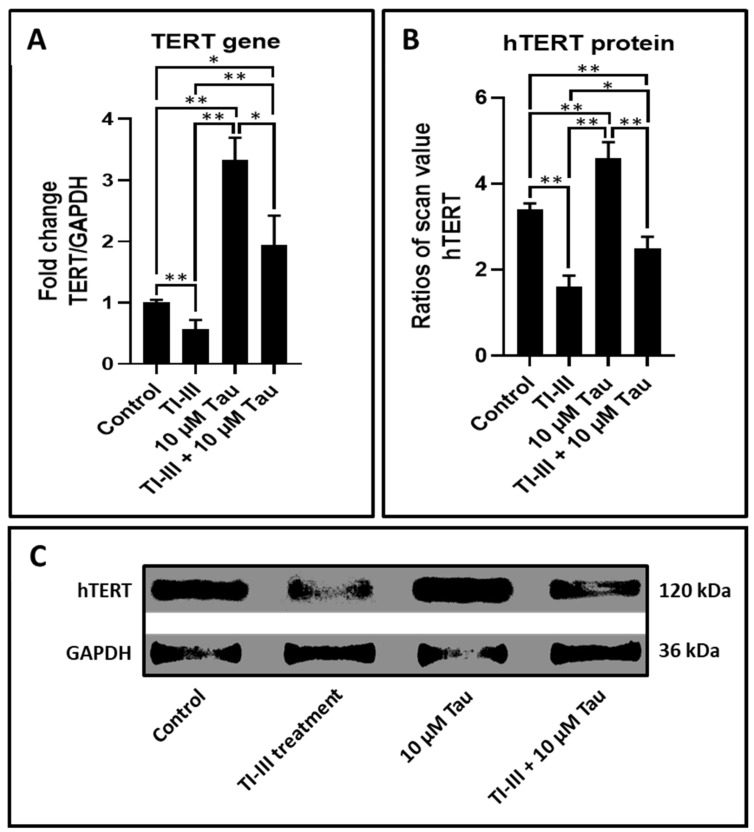
Taurine upregulates gene expression of TERT and protein levels of hTERT in DPSCs. (**A**) Comparative gene expression analysis of TERT with and without taurine treated DPSCs and telomerase inhibited DPSCs. (**B**,**C**) Whole cell extracts were evaluated by using the western blot method to decide the protein expression levels of hTERT in DPSCs. The graphs show the ratios of band densities of hTERT. ns: not significant, * *p* < 0.05, ** *p* < 0.01. Tau: Taurine, TI-III: telomerase inhibitor III, TERT/hTERT: human telomerase reverse transcriptase.

DPSCs on treatment with taurine demonstrated increased expression of the TERT gene in comparison with untreated cells. Additionally, taurine augmented the TERT expression in telomerase inhibitor treated DPSCs (Table 5 and Figure 3A).

The higher levels of protein expression of hTERT in DPSCs treated with taurine specified the functional augmentation of TERT gene expression (Table 6 and Figure 3B,C).

Taurine treatment promotes chondrogenesis in DPSCs and telomerase inhibited DPSCs and upregulates expression of chondrogenesis related genes.

**Figure 4:**
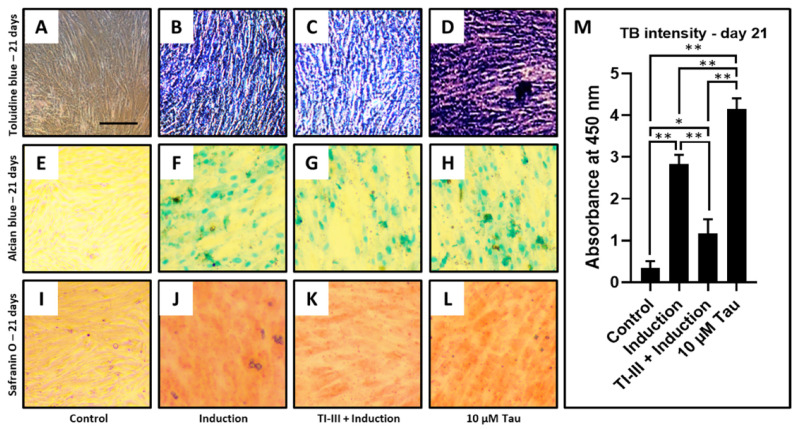
Chondrogenic differentiation of DPSCs and quantification of toluidine blue intensity. Chondrogenic differentiation was induced in DPSCs with and without treatment with telomerase inhibitor and DPSCs treated with taurine alone were assessed for chondrogenesis. Functional staining was done with (**A**–**D**) toluidine blue, (**E**–**H**) Alcian blue, and (**I**–**L**) safranin O. Scale bar = 100 μm, (**M**) Comparative quantification of toluidine blue intensity day 21. * *p* < 0.05, ** *p* < 0.01. Induction: Chondrogenic differentiation, Tau: Taurine, TI-III: Telomerase Inhibitor III, TB: Toluidine blue.

Chondrogenic differentiation was induced in DPSCs and telomerase inhibited DPSCs, and the treatment of 10 µM taurine was given to the DPSCs (Figure 4A–L). It was observed that the differentiation of DPSCs into chondrocytes was significantly inhibited with telomerase inhibitor treatment (Figure 4C,M), but taurine alone was shown to promote chondrogenesis by toluidine blue, Alcian blue, and safranin O staining (Table 7 and Figure 4D,H,L). Additionally, toluidine blue intensity quantification was checked and the same trend was observed (Figure 4M).

**Figure 5:**
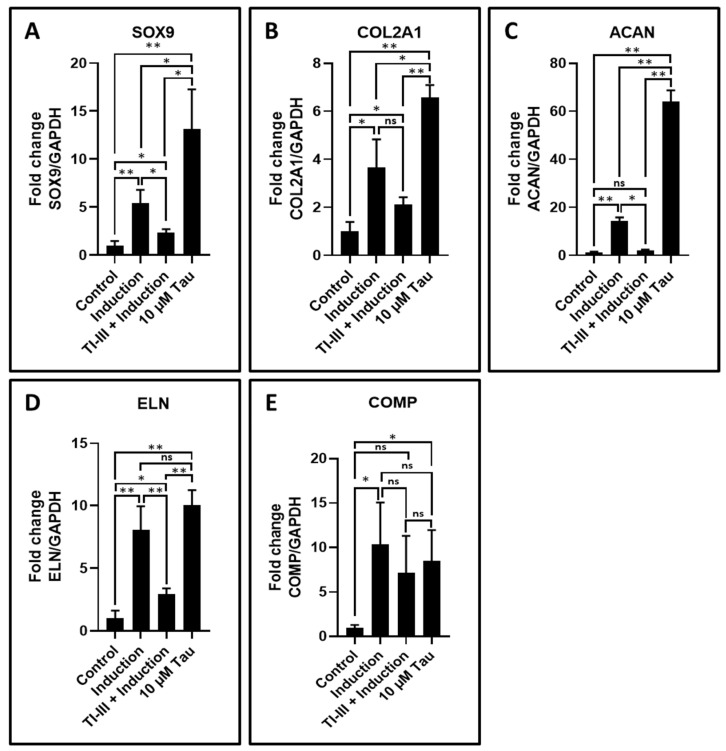
Chondrogenic differentiation of DPSCs and the analysis of chondrogenesis related gene expression by quantitative RT-qPCR. (**A**–**E**) Comparative gene expression analysis of chondrogenesis-related genes SOX9, COL2A1, ACAN, ELN, and COMP in chondrogenesis induced DPSCs with and without treatment with telomerase inhibitor and DPSCs treated with taurine alone. ns: not significant, * *p* < 0.05, ** *p* < 0.01. Induction: chondrogenic differentiation, Tau: taurine, TI-III: telomerase Inhibitor III, SOX9: SRY-box transcription factor 9, COL2A1: collagen, type II, alpha 1, ACAN: aggrecan, ELN: elastin, COMP: cartilage oligomeric matrix protein.

Chondrogenesis related genes were assessed in the control, induction, TI-III + induction, and 10 μM taurine treated DPSCs. SOX9, COL2A1, ACAN, and ELN showed upregulated expression with only taurine treatment and the expression levels of these genes were significantly higher than induced DPSCs (Table 8 and Figure 5A–D). The expression levels of these genes were significantly downregulated in the telomerase inhibited induced DPSCs, whereas expression level of COMP was found to be increased in induced and taurine treated DPSCs, but no significant difference was observed between these two groups (Table 8 and Figure 5E).

Taurine treatment promotes chondrogenesis in DPSCs and show expression of chondrogenesis related proteins SOX-9 and aggrecan.

**Figure 6:**
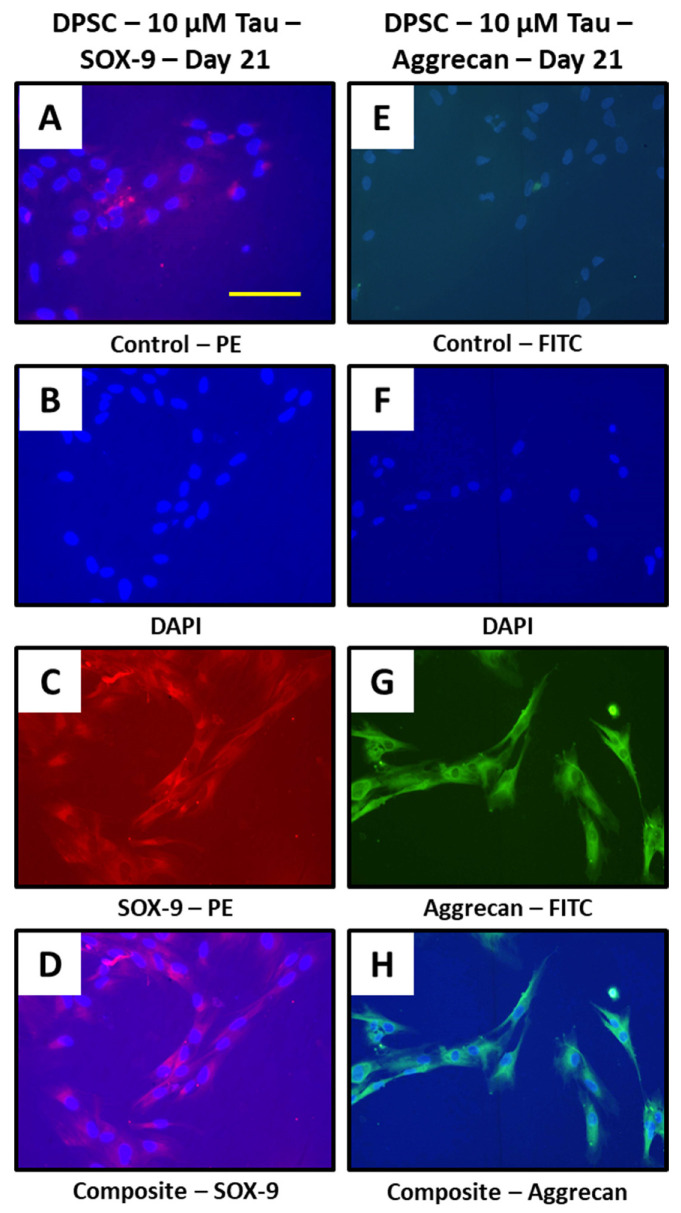
Taurine treatment to DPSCs and immunostaining. (**A**–**H**) DPSCs treated with taurine alone were assessed for chondrogenesis related proteins SOX-9 and aggrecan at day 21. Scale bar = 200 μm. Tau: taurine.

Chondrogenesis related proteins SOX-9 and aggrecan were assessed in 10 μM taurine treated DPSCs at day 21. Both proteins showed expression by immunostaining (Figure 6A–H).

## 4. Discussion

The present in vitro study highlights the use of taurine as a stimulant for chondrogenic differentiation of DPSCs. It is well known that DPSCs represent a population of mesenchymal stem cells of dental origin. These are isolated from the human dental pulp. This is an easily accessible source and can be efficiently harvested and expanded for use. The potential of DPSCs and their secretome in regeneration and differentiation into an array of cell types has been recently documented [45]. From these type of studies, it is apparent that the regenerative potential of DPSCs is far superior compared to bone marrow mesenchymal stem cells. Hence, our choice of the type of stem cell used for the present study can be justified from this viewpoint. A confirmation of the surface markers of the DPSCs used in the present study revealed that they expressed CD73, CD90, and CD105 and did not express CD34, CD45, and HLA-DR. These findings are confirmatory that the cells are of mesenchymal origin and do not express any hematopoietic markers. After an initial characterization of the DPSCs, they were subjected to an MTT assay with taurine administration in order to assess the cytotoxic effects of taurine and to determine the ideal taurine concentration for further experimentation. Data revealed that none of the concentrations of taurine affected cell viability for up to 48 h. A concentration of 10 µM of taurine was fixed for all further experiments.

With regard to telomerase gene (TERT) expression in DPSCs under the influence of taurine, it was found that the addition of telomerase inhibitor to the cells significantly lowered TERT expression. However, the addition of taurine increased the TERT expression significantly. It was observed that when there was a simultaneous addition of taurine and the telomerase inhibitor, there was a decrease in TERT expression in the DPSCs, however, this value was significantly higher than the group with only the addition of the telomerase inhibitor. These findings go toward proving the proliferative induction caused by taurine on the DPSCs. The TERT gene encodes the hTERT protein, which is an active component of the telomerase enzyme that is responsible for the restoration of telomeric length in eukaryotic cells. It is noteworthy that an increase in telomeric length implies enhanced cellular longevity and proliferation potential [40]. Hence, the present study findings point to an increase in proliferative activity of the DPSCs under the influence of taurine. To confirm the genetic findings at the protein level, a western blot analysis was done, which confirmed that there was an elevation in hTERT protein levels following taurine addition to DPSC.

An increase in proliferative activity of the DPSCs under the influence of taurine also implicates an increase in synthetic activity. In this regard, it was assessed whether taurine caused chondrogenic differentiation of the DPSCs. It was found that there was an increase in the SOX9, COL2A1, ACAN, and ELN genes specific for chondrocytes in the DPSCs treated with taurine. The values of the gene expression were also significantly higher in the taurine only group compared to the induction group. There was a significant reduction in the expression of the above-mentioned genes in the telomerase inhibited induced DPSCs. The above findings shed light on the fact that taurine has a dual role in increasing telomerase activity and an enhancing effect on the cartilage specific gene expression. To confirm if cartilage specific markers were expressed by the DPSCs under taurine influence, a quantification of glycosamino glycan content was undertaken. The results in this regard revealed that taurine treated DPSCs produced significantly higher amounts of glycosaminoglycans. It is evident from the findings of the study that taurine can act as a driving force in enhancing telomerase activity in DPSC by its stimulatory effects on the hTERT gene and protein expression.

Taurine was also found to enhance chondrogenesis related gene expression in DPSCs by driving them toward a chrondrocyte phenotype, as evidenced by upregulated glycosaminoglycan production. A similar effect of taurine on enhancing chondrogenic differentiation of umbilical cord derived mesenchymal stem cells has already been documented. In this study, it was observed that taurine significantly upregulated collagen type 2, aggrecan, and SOX 9 gene expression and caused increased glycosaminoglycan synthesis in the cells [46]. The present study results are in line with the above study with regard to chondrocytic differentiation. Another in vitro study has also highlighted the superior properties of taurine with regard to cartilage repair [47]. The present study is novel compared to the previous studies performed in this area of research as it was performed on DPSCs, which has not been done before. Additionally, the measurement of the hTERT gene and protein expression is a unique finding of the study and corroborates previous studies that have ascertained that taurine increases proliferation rates in chondrocytes [48,49,50].

## 5. Conclusions

It is evident that taurine is a largely biocompatible and non-cytotoxic small molecule that can be used as a biologic modifier along with DPSCs to promote cartilage repair. It can also be applied onto scaffolds that are used in cartilage regeneration procedures. The limitations of the present study are the fact that it is an in vitro study with a single cell type. Additionally, in the present study, the cells were not seeded onto the appropriate scaffolds with regenerative properties that could aid in clinical application. Future in vivo studies in animal models followed by clinical trials have to be performed to explore the regenerative property of taurine induced DPSCs, thereby aiding in the management of several degenerative disorders of cartilage including inflammatory arthropathies such as rheumatoid arthritis, systemic lupus erythematosus; chondrocalcinosis; relapsing polychondritis; osteochondritis dissecans; and developmental disorders like achondroplasias, chrondrosdysplasias, and osteoarthritis.

## Figures and Tables

**Table 1:** Effect of taurine on chondrogenesis—Experimental groups.

Experimental Group	Treatment
Control	-
Induction	Chondrogenic induction media treatment
TI-III + Induction	Telomerase inhibitor + Chondrogenic induction media treatment
10 µM Tau	10 µM taurine treatment

**Table 2:** Effect of taurine on TERT gene and protein expression—Experimental groups.

Experimental Group	Treatment
Control	-
TI-III	Telomerase inhibitor treatment
10 µM Tau	10 µM taurine treatment
TI-III + 10 µM Tau	Telomerase inhibitor + 10 µM taurine treatment

**Table 3:** List of primers.

Gene	Forward Primer	Reverse Primer
TERT	5′-GCC GAT TGT GAA CAT GGA CTA CG-3′	5′-GCT CGT AGT TGA GCA CGC TGA A-3′
SOX9	5′-GCC GAA AGC GGG CTC GAA AC-3′	5′-AAA AGT GGG GGC GCT TGC ACC-3′
COL2A1	5′-CCT CCA GGT CTT CAG GGA AT-3′	5′-AGG AGG TCC AAC TTC TCC CT-3′
ACAN	5′-GCG AGT TGT CAT GGT CTG AA-3′	5′-TTC TTG GAG AAG GGA GTC CA-3′
ELN	5′-GGT TGT GTC ACC AGA AGC AGC T-3′	5′-CCG TAA GTA GGA ATG CCT CCA AC-3′
COMP	5′-GGA GAT GCT TGT GAC AGC GAT C-3′	5′-TGA GTC CTC CTG GGC ACT GTT A-3′
GAPDH	5′-GTC TCC TCT GAC TTC AAC AGC G-3′	5′-ACC ACC CTG TTG CTG TAG CCA A-3′

**Table 4:** MTT assay—absorbance at 570 nm.

Concentration	MTT—48 h	MTT—7 Days
Control	0.46 ± 0.029	1.28 ± 0.42
1 µM Tau	0.48 ± 0.073	1.63 ± 0.10
2.5 µM Tau	0.41 ± 0.09	1.62 ± 0.17
5 µM Tau	0.53 ± 0.03	1.59 ± 0.31
10 µM Tau	0.57 ± 0.034	1.30 ± 0.29
25 µM Tau	0.34 ± 0.10	0.27 ± 0.09
50 µM Tau	0.25 ± 0.15	0.09 ± 0.02

**Table 5:** Relative gene expression of TERT (fold change).

Gene	Control	TI-III	10 µM Tau	TI-III + 10 µM Tau
TERT	1.00 ± 0.05	0.57 ± 0.15	3.33 ± 0.36	1.95 ± 0.47

**Table 6:** hTERT protein expression.

Gene	Control	TI-III	10 µM Tau	TI-III + 10 µM Tau
hTERT	3.42 ± 0.14	1.61 ± 0.26	4.63 ± 0.36	2.50 ± 0.26

**Table 7:** Toluidine blue (TB) intensity analysis.

Concentration	TB Intensity—Day 21
Control	0.35 ± 0.15
Induction	2.83 ± 0.21
TI-III + Induction	1.16 ± 0.34
10 µM Tau	4.14 ± 0.25

**Table 8:** Relative gene expression of chondrogenesis related genes (fold change).

Gene	Control	Induction	TI-III + Induction	10 µM Tau
SOX9	1.00 ± 0.48	5.38 ± 1.43	2.37 ± 0.34	13.15 ± 4.11
COL2A1	1.00 ± 0.39	3.65 ± 1.18	2.13 ± 0.28	6.58 ± 0.51
ACAN	1.00 ± 0.48	14.34 ± 1.45	2.03 ± 0.35	63.96 ± 4.72
ELN	1.00 ± 0.62	8.09 ± 1.86	2.96 ± 0.45	10.05 ± 1.21
COMP	1.00 ± 0.31	10.33 ± 4.74	7.21 ± 4.10	8.47 ± 3.48

## References

[1] Buckwalter J.A., Mankin H.J. (1998). Articular cartilage repair and transplantation. Arthritis Rheum..

[2] Bentley G., Biant L.C., Carrington R.W.J., Akmal M., Goldberg A., Williams A.M., Skinner J.A., Pringle J. (2003). A prospective, randomised comparison of autologous chondrocyte implantation versus mosaicplasty for osteochondral defects in the knee. J. Bone Jt. Surg. Br..

[3] Knutsen G., Engebretsen L., Ludvigsen T.C., Drogset J.O., Grøntvedt T., Solheim E., Strand T., Roberts S., Isaksen V., Johansen O. (2004). Autologous chondrocyte implantation compared with microfracture in the knee. A randomized trial. J. Bone Jt. Surg. Am..

[4] Tatullo M., Spagnuolo G., Codispoti B., Zamparini F., Zhang A., Esposti M., Aparicio C., Rengo C., Nuzzolese M., Manzoli L. (2019). PLA-Based Mineral-Doped Scaffolds Seeded with Human Periapical Cyst-Derived MSCs: A Promising Tool for Regenerative Healing in Dentistry. Materials.

[5] Scheibe F., Ladhoff J., Huck J., Grohmann M., Blazej K., Oersal A., Baeva N., Seifert M., Priller J. (2012). Immune effects of mesenchymal stromal cells in experimental stroke. J. Cereb. Blood Flow Metab..

[6] Trounson A., McDonald C. (2015). Stem Cell Therapies in Clinical Trials: Progress and Challenges. Cell Stem Cell.

[7] Boese A.C., Le Q.-S.E., Pham D., Hamblin M.H., Lee J.-P. (2018). Neural stem cell therapy for subacute and chronic ischemic stroke. Stem Cell Res. Ther..

[8] Leong W.K., Lewis M.D., Koblar S.A. (2013). Concise review: Preclinical studies on human cell-based therapy in rodent ischemic stroke models: Where are we now after a decade?. Stem Cells.

[9] Prockop D.J., Brenner M., Fibbe W.E., Horwitz E., Le Blanc K., Phinney D.G., Simmons P.J., Sensebe L., Keating A. (2010). Defining the risks of mesenchymal stromal cell therapy. Cytotherapy.

[10] Lees J.S., Sena E.S., Egan K.J., Antonic A., Koblar S.A., Howells D.W., Macleod M.R. (2012). Stem cell-based therapy for experimental stroke: A systematic review and meta-analysis. Int. J. Stroke.

[11] Savitz S.I. (2015). Developing Cellular Therapies for Stroke. Stroke.

[12] Kalladka D., Muir K.W. (2014). Brain repair: Cell therapy in stroke. Stem Cells Cloning.

[13] Meng H., Hu L., Zhou Y., Ge Z., Wang H., Wu C., Jin J. (2020). A Sandwich Structure of Human Dental Pulp Stem Cell Sheet, Treated Dentin Matrix, and Matrigel for Tooth Root Regeneration. Stem Cells Dev..

[14] Fujii Y., Kawase-Koga Y., Hojo H., Yano F., Sato M., Chung U., Ohba S., Chikazu D. (2018). Bone regeneration by human dental pulp stem cells using a helioxanthin derivative and cell-sheet technology. Stem Cell Res. Ther..

[15] Marrelli M., Codispoti B., Shelton R.M., Scheven B.A., Cooper P.R., Tatullo M., Paduano F. (2018). Dental Pulp Stem Cell Mechanoresponsiveness: Effects of Mechanical Stimuli on Dental Pulp Stem Cell Behavior. Front. Physiol..

[16] Pedroni A.C.F., Sarra G., de Oliveira N.K., Moreira M.S., Deboni M.C.Z., Marques M.M. (2019). Cell sheets of human dental pulp stem cells for future application in bone replacement. Clin. Oral Investig..

[17] Monteiro N., Smith E.E., Angstadt S., Zhang W., Khademhosseini A., Yelick P.C. (2016). Dental cell sheet biomimetic tooth bud model. Biomaterials.

[18] Casado-Díaz A., Anter J., Dorado G., Quesada-Gómez J.M. (2016). Effects of quercetin, a natural phenolic compound, in the differentiation of human mesenchymal stem cells (MSC) into adipocytes and osteoblasts. J. Nutr. Biochem..

[19] Ballini A., Boccaccio A., Saini R., Van Pham P., Tatullo M. (2017). Dental-Derived Stem Cells and Their Secretome and Interactions with Bioscaffolds/Biomaterials in Regenerative Medicine: From the In Vitro Research to Translational Applications. Stem Cells Int..

[20] Bouckenooghe T., Remacle C., Reusens B. (2006). Is taurine a functional nutrient?. Curr. Opin. Clin. Nutr. Metab. Care.

[21] Birdsall T.C. (1998). Therapeutic applications of taurine. Altern. Med. Rev..

[22] Warskulat U., Heller-Stilb B., Oermann E., Zilles K., Haas H., Lang F., Häussinger D. (2007). Phenotype of the taurine transporter knockout mouse. Methods Enzymol..

[23] Klusa V., Klimaviciusa L., Duburs G., Poikans J., Zharkovsky A. (2006). Anti-neurotoxic effects of tauropyrone, a taurine analogue. Adv. Exp. Med. Biol..

[24] Petrovic L., Schlegel K.A., Ries J., Park J., Diebel E., Schultze-Mosgau S., Wiltfang J. (2003). In Vitro effect of taurolidine on squamous cell carcinoma in the oral cavity. Mund. Kiefer. Gesichtschir..

[25] Marcinkiewicz J., Kurnyta M., Biedroń R., Bobek M., Kontny E., Maśliński W. (2006). Anti-inflammatory effects of taurine derivatives (taurine chloramine, taurine bromamine, and taurolidine) are mediated by different mechanisms. Adv. Exp. Med. Biol..

[26] Zeybek A., Sağlam B., Cikler E., Cetinel S., Ercan F., Sener G. (2007). Taurine ameliorates stress-induced degeneration of the urinary bladder. Acta Histochem..

[27] Zeybek A., Ercan F., Cetinel S., Cikler E., Sağlam B., Sener G. (2006). Taurine ameliorates water avoidance stress-induced degenerations of gastrointestinal tract and liver. Dig. Dis. Sci..

[28] Zeybek A., Sağlam B., Cikler E., Cetinel S., Ercan F., Sener G. (2006). Protective effects of taurine on protamine sulfate induced bladder damage. World J. Urol..

[29] Liao X.-B., Zhou X.-M., Li J.-M., Tan Z.-P., Liu L.-M., Zhang W., Tan H., Lu Y., Yuan L.-Q. (2007). Taurine transporter is expressed in vascular smooth muscle cells. Amino Acids.

[30] Allard M.L., Jeejeebhoy K.N., Sole M.J. (2006). The management of conditioned nutritional requirements in heart failure. Heart Fail. Rev..

[31] Gupta R.C., Win T., Bittner S. (2005). Taurine analogues; a new class of therapeutics: Retrospect and prospects. Curr. Med. Chem..

[32] Parcell S. (2002). Sulfur in human nutrition and applications in medicine. Altern. Med. Rev..

[33] Guizouarn H., Motais R., Garcia-Romeu F., Borgese F. (2000). Cell volume regulation: The role of taurine loss in maintaining membrane potential and cell pH. J. Physiol..

[34] Kong W.X., Chen S.W., Li Y.L., Zhang Y.J., Wang R., Min L., Mi X. (2006). Effects of taurine on rat behaviors in three anxiety models. Pharmacol. Biochem. Behav..

[35] Soyka M., Roesner S. (2006). New pharmacological approaches for the treatment of alcoholism. Expert Opin. Pharmacother..

[36] Trip J., Drost G., van Engelen B.G.M., Faber C.G. (2006). Drug treatment for myotonia. Cochrane Database Syst. Rev..

[37] Yu X., Xu Z., Mi M., Xu H., Zhu J., Wei N., Chen K., Zhang Q., Zeng K., Wang J. (2008). Dietary taurine supplementation ameliorates diabetic retinopathy via anti-excitotoxicity of glutamate in streptozotocin-induced Sprague-Dawley rats. Neurochem. Res..

[38] Grimble R.F. (2006). The effects of sulfur amino acid intake on immune function in humans. J. Nutr..

[39] Tan B., Jiang D.-J., Huang H., Jia S.-J., Jiang J.-L., Hu C.-P., Li Y.-J. (2007). Taurine protects against low-density lipoprotein-induced endothelial dysfunction by the DDAH/ADMA pathway. Vascul. Pharmacol..

[40] Liu Q., Lu Z., Wu H., Zheng L. (2015). Chondroprotective effects of taurine in primary cultures of human articular chondrocytes. Tohoku J. Exp. Med..

[41] Wei F., Qu C., Song T., Ding G., Fan Z., Liu D., Liu Y., Zhang C., Shi S., Wang S. (2012). Vitamin C treatment promotes mesenchymal stem cell sheet formation and tissue regeneration by elevating telomerase activity. J. Cell. Physiol..

[42] Patil V.R., Kharat A.H., Kulkarni D.G., Kheur S.M., Bhonde R.R. (2018). Long term explant culture for harvesting homogeneous population of human dental pulp stem cells. Cell Biol. Int..

[43] Bergholt N.L., Lysdahl H., Lind M., Foldager C.B. (2019). A Standardized Method of Applying Toluidine Blue Metachromatic Staining for Assessment of Chondrogenesis. Cartilage.

[44] Livak K.J., Schmittgen T.D. (2001). Analysis of Relative Gene Expression Data Using Real-Time Quantitative PCR and the 2−ΔΔCT Method. Methods.

[45] Kumar A., Kumar V., Rattan V., Jha V., Pal A., Bhattacharyya S. (2017). Molecular spectrum of secretome regulates the relative hepatogenic potential of mesenchymal stem cells from bone marrow and dental tissue. Sci. Rep..

[46] Yao X., Huang H., Li Z., Liu X., Fan W., Wang X., Sun X., Zhu J., Zhou H., Wei H. (2017). Taurine Promotes the Cartilaginous Differentiation of Human Umbilical Cord-Derived Mesenchymal Stem Cells in Vitro. Neurochem. Res..

[47] Pellegrino L., Cocchiola R., Francolini I., Lopreiato M., Piozzi A., Zanoni R., Scotto d’Abusco A., Martinelli A. (2017). Taurine grafting and collagen adsorption on PLLA films improve human primary chondrocyte adhesion and growth. Colloids Surf. B Biointerfaces.

[48] Jeon S.-H., Lee M.-Y., Kim S.-J., Joe S.-G., Kim G.-B., Kim I.-S., Kim N.-S., Hong C.-U., Kim S.-Z., Kim J.-S. (2007). Taurine increases cell proliferation and generates an increase in [Mg2+]i accompanied by ERK 1/2 activation in human osteoblast cells. FEBS Lett..

[49] Hernández-Benítez R., Pasantes-Morales H., Saldaña I.T., Ramos-Mandujano G. (2010). Taurine stimulates proliferation of mice embryonic cultured neural progenitor cells. J. Neurosci. Res..

[50] Hernández-Benítez R., Ramos-Mandujano G., Pasantes-Morales H. (2012). Taurine stimulates proliferation and promotes neurogenesis of mouse adult cultured neural stem/progenitor cells. Stem Cell Res..

